# Quantifying and Exploiting the Age Dependence in the Effect of Supplementary Food for Child Undernutrition

**DOI:** 10.1371/journal.pone.0099632

**Published:** 2014-06-26

**Authors:** Milinda Lakkam, Stefan Wager, Paul H. Wise, Lawrence M. Wein

**Affiliations:** 1 Institute for Computational and Mathematical Engineering, Stanford University, Stanford, California, United States of America; 2 Statistics Department, Stanford University, Stanford, California, United States of America; 3 School of Medicine, Stanford University, Stanford California, United States of America; 4 Graduate School of Business, Stanford University, Stanford, California, United States of America; CUNY, United States of America

## Abstract

Motivated by the lack of randomized controlled trials with an intervention-free control arm in the area of child undernutrition, we fit a trivariate model of weight-for-age z score (WAZ), height-for-age z score (HAZ) and diarrhea status to data from an observational study of supplementary feeding (100 kCal/day for children with WAZ 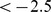) in 17 Guatemalan communities. Incorporating time lags, intention to treat (i.e., to give supplementary food), seasonality and age interactions, we estimate how the effect of supplementary food on WAZ, HAZ and diarrhea status varies with a child’s age. We find that the effect of supplementary food on all 3 metrics decreases linearly with age from 6 to 20 mo and has little effect after 20 mo. We derive 2 food allocation policies that myopically (i.e., looking ahead 2 mo) minimize either the underweight or stunting severity – i.e., the sum of squared WAZ or HAZ scores for all children with WAZ or HAZ 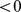. A simulation study based on the statistical model predicts that the 2 derived policies reduce the underweight severity (averaged over all ages) by 13.6–14.1% and reduce the stunting severity at age 60 mo by 7.1–8.0% relative to the policy currently in use, where all policies have a budget that feeds 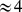
% of children. While these findings need to be confirmed on additional data sets, it appears that in a low-dose (100 kCal/day) supplementary feeding setting in Guatemala, allocating food primarily to 6–12 mo infants can reduce the severity of underweight and stunting.

## Introduction

With over 3 M deaths per year of children under 5 years attributable to undernutrition [1] and the level of food aid far less than required [2], [3], it is vital to optimally allocate food to the appropriate children in the appropriate amounts. To address this problem in a rigorous manner requires knowledge about three key aspects [4]: (i) the evolution of weight and height (and perhaps disease) of children under 5 years in the absence of food aid; (ii) the impact that weight and height (and perhaps other factors such as age, sex and disease) have on morbidity and mortality, and (iii) the impact of supplementary or therapeutic food on weight and height (and perhaps other factors such as disease).

A bivariate statistical model of weight and height can be constructed from a longitudinal data set [4] to address (i), and logistic regression [4], [5] or proportional hazards [6] models can estimate the impact of anthropometric measurements on mortality to address (ii). The ideal approach to (iii) would use longitudinal measurements of weight and height from a large randomized control trial with a control arm that does not receive any food. There appears to be only one study that possesses these characteristics for therapeutic food [7] and no studies involving supplementary feeding [8]. As a result, very little is known about how the impact of food on weight and height is influenced by a child’s age, sex and pre-intervention weight, height and presence of disease, and the amount of therapeutic or supplementary food consumed. Furthermore, the ethical issues inherent in using a control group that does not receive food suggests that there may not be any large randomized control trials with intervention-free arms performed in the future.

Consequently, the most promising approach to making headway on aspect (iii) is to jointly estimate aspects (i) and (iii) – i.e., fit a statistical model of weight and height in the presence of nonrandom food aid – using observational or dose-response studies of nutrition programs that do not include intervention-free control arms. In this study, we make a first step in this direction and analyze data from a nutrition program in Guatemala, where every child under 5 years in 17 villages has his weight, height and diarrhea status (the number of days in the previous week with diarrhea) measured every 2 mo, and children with weight-for-age z score (WAZ) less than −2.5 receive supplementary food of 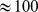 kCal/day. We use the WAZ = −2.5 threshold to cast the nutrition program as a natural experiment – where children with WAZ just above −2.5 (who do not receive treatment) act as a control group for the children with WAZ just below **−**2.5 (who do receive treatment) – and analyze it via a regression discontinuity design [9], which is a design where the assignment of an intervention is based on the value of an observed covariate lying below (or above) a fixed threshold. By comparing observations that are just above and just below WAZ = −2.5, the regression discontinuity design allows us to estimate the average treatment effect. In econometrics, statistics and related fields, regression discontinuity designs have become the preferred approach for evaluating the effects of intervention in a setting such as ours, where randomization is not possible [9]. More specifically, a generalized additive model (GAM) [10] is used to predict future (i.e., 2 mo hence) WAZ, height-for-age z score (HAZ) and diarrhea status (because deaths from wasting do not occur in this setting, we focus on WAZ and HAZ instead of weight-for-height z score, WHZ) in terms of current and past values of (WAZ,HAZ,diarrhea) along with seasonal and age-interaction terms. This statistical model allows us to isolate how age influences the impact that supplementary food has on WAZ, HAZ and diarrhea status. We use these statistical results to derive 2 allocation policies that minimize the underweight (or stunting) severity, i.e., the sum of squared expected WAZ (or HAZ) scores among children with WAZ 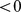
(or HAZ 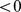), 2 mo into the future. We build a simulation model based on our GAM that compares the derived allocation policies to the allocation policy that is currently used in the nutrition program in Guatemala, and to the counterfactual of no supplementary food.

## Materials and Methods

### Data

Our data set tracks 2125 children (1047 boys, 1078 girls) from 17 communities participating in a nutrition program in Guatemala from November 2009 to April 2012. Every 2 months, children from birth to age 60 mo had their WAZ, HAZ – both measured according to World Health Organization (WHO) child growth standards [11] – and the number of days with diarrhea in the previous week recorded. The nutrition program provided supplementary food to all children with WAZ 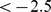. Children over 12 mo received 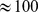 kCal/day of Incaparina (a supplement made from maize and soy flours) and sugar, and children between 6–12 mo received half this amount and – for children with inadequate or no breastfeeding despite promoter efforts to support breastfeeding until at least 1 year of age –2 400-gm cans of powdered infant formula (Nestle NAN Pro 1 or 2, made of whey and casein milk proteins) per mo. The Stanford Institutional Review Board, which includes members with medical and social science expertise, and the Review Committee of the Hospital Obras Sociales de Monsignor Gregorio Schaffer, San Lucas Toliman reviewed and approved this study prior to study initiation. Informed consent in Spanish and/or the local indigenous language was obtained from a parent/guardian for all children at enrollment. All patient information was anonymized and de-identified prior to analysis.

### Generalized Additive Model

For boys and girls, we build separate GAMs for the evolution of (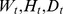) for age *t* varying from 1 to 60 mo, which are the WAZ, HAZ and the number of days with diarrhea in the previous week for a child of age *t* mo (see Table 1 for a description of most of the variables). Define the Intention To Treat (ITT) indicator variable 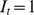 if 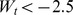 and 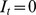 otherwise, and let *M_ t_* be the month of the year when a child is of age *t*. Let 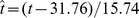 be the standardized age, where 31.76 mo and 15.74 mo are the mean and standard deviation of the ages among all entries in the data set.

**Table 1:** The estimated parameters for boys from the GAM.

		Estimating *W_t_*_+2_−*W_t_*			Estimating *H_t_*_+2_−*H_t_*			Estimating *D_t_*_+2_		
Variable	Description	Est.	S.E.	*p*	Est.	S.E.	*p*	Est.	S.E.	*p*
	Intercept	−0.230	0.018	0.000	−0.907	0.025	0.000	−0.751	0.081	0.000
*W_t_*	WAZ	−0.095	0.007	0.000	0.124	0.009	0.000	−0.184	0.039	0.000
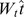	WAZ 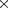 age	0.014	0.006	0.026	−0.068	0.009	0.000	−0.056	0.036	0.115
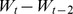	WAZ increment	−0.256	0.011	0.000	−0.000	0.016	0.981	0.183	0.066	0.006
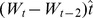	WAZ incr. 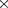 age	−0.090	0.010	0.000	0.021	0.013	0.116	0.084	0.055	0.122
*H_t_*	HAZ	0.038	0.006	0.000	−0.177	0.008	0.000	−0.007	0.033	0.835
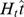	HAZ 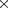 age	0.006	0.005	0.280	0.092	0.007	0.000	0.068	0.030	0.024
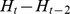	HAZ increment	−0.020	0.009	0.030	−0.205	0.013	0.000	0.063	0.053	0.236
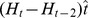	HAZ incr. 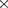 age	−0.007	0.008	0.375	−0.022	0.011	0.036	−0.041	0.042	0.337
*D_t_*	Diarrhea	0.003	0.003	0.387	−0.003	0.004	0.429	0.102	0.014	0.000
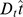	Diarrhea 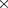 age	0.014	0.003	0.000	0.007	0.004	0.091	−0.021	0.013	0.100

For each variable, we give the estimated coefficient (Est.), the standard error (S.E.) and the p-value.

An analysis of GAM results with varying amounts of history suggests that we need to maintain 4 mo of history; i.e., if we are trying to estimate a child’s 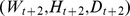 then we need to use 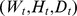 and 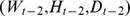. To ease the interpretation of our GAM coefficients, we let the response variables be 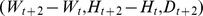. We fit 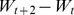 and 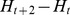 by least squares and model *D_t_* by a Poisson random variable. We let the link functions be the identity function for 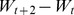 and 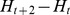 and the natural logarithm for 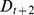. The predictor variables include the current values (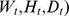 and their age-interaction terms 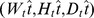, the most recent increments 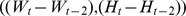 and their age interaction terms 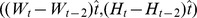, as well as seasonal functions 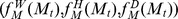 for the month of the year, age functions (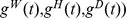 in the absence of supplementary food, and age functions (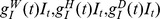 due to the ITT. The seasonal functions are modeled as periodic cubic splines and the age functions are modeled as cubic smoothing splines. We denote the sample covariance matrix calculated from the residuals of the GAM by 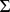. The 3 GAM equations are stated with their resulting coefficients in §1 of File S1.

To make the most use of the available data without imputing any missing data, we include every set of 3 consecutive measurements by assuming they occur at ages *t* –2, *t* and *t* +2 for some *t*. We also discard as outliers any z scores outside the range of [**−**5,5].

### Proposed Allocation Policies

Because of the difficulty of solving a dynamic program with a system state that is the joint population-wide probability density function (PDF) of (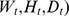, we derive 2 derived policies – referred to as the derived WAZ policy and the derived HAZ policy – that estimate every child’s 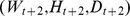 given all the information known at age *t*, and minimizes the sum of 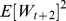 (or 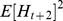) over all children with 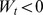
(or 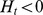), subject to a constraint on the amount of supplementary food allocated. As in [12], this objective captures moderate and mild forms of undernutrition and also incorporates the fact that more severe undernutrition leads to more severe consequences; however, we use 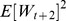 rather than 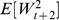 so that the proposed policy is relatively simple.

Let 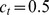 if 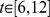 and 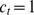 if 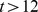, which represents the normalized amount of supplementary food consumed by a child at age *t*. The allocation policy that solves this constrained optimization problem prioritizes all children with 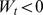 by 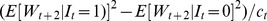 (with smaller values getting higher priority), and allocates the food until it is gone (§2 of File S1).

### Simulation Model

The simulation model tracks 1 M children of each sex from age 6 to 60 mo. A child’s initial 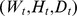 at 6 mo is randomly sampled with replacement from all children of the same sex in the data set when they were 6 mo. At each 2-mo time step, the values of 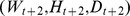 are calculated using the GAM and then adding an additive trivariate noise term that is normally distributed with mean zero and covariance matrix 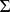 (and rounding the value of 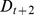 to the nearest integer).

We consider the 5 policies described in Table 2. All policies except the no food policy allocate the same amount of food, which is dictated by the amount of food allocated under the current policy (i.e., 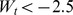). This food budget is 1.2 (0.7) units of food per boy (girl) over a child’s 27 feeding opportunities, which corresponds to 4.7% of children being fed at any given time. We do a 1-dimensional search for thresholds (for the quantities being prioritized in Table 2) for the simple WAZ policy and the 2 derived policies so that the amount of food allocated by these policies is equal to this food budget. The thresholds for the simple WAZ policy are **−**1.8 for boys (i.e., a boy under 2 yr receives food if 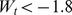) and **−**1.7 for girls, the thresholds for the derived WAZ policy are **−**0.082 for boys (i.e., a boy receives food if 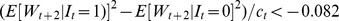) and **−**0.138 for girls, and the thresholds for the derived HAZ policy are **−**0.44 for boys and **−**0.56 for girls.

**Table 2:** Food allocation policies.

Name of Policy	Children Receiving Food	Severity Index	
		Underweight	Stunting
No Food	None	1.87	5.44
Current	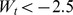	1.84	5.50
Simple WAZ	Under 2 yr and prioritized by *W_t_*	1.77	5.35
Derived WAZ	Prioritized by 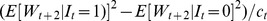	1.59	5.11
Derived HAZ	Prioritized by 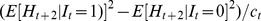	1.58	5.06

A description of the food allocation policies and their severity indices for boys, which measure the average of squared shortfalls below the reference median (i.e., zero) for WAZ averaged over all 28 measured ages (underweight) and for HAZ at age 60 mo (stunting).

For each policy and various values of 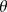, we report 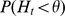 and 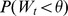 for all 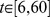 mo. We also compute 2 variants of the severity index considered in [12], the average of 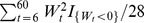 over all children and the average of 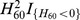, where 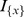 is the indicator function of the event *x*. These quantities are the average squared shortfalls below the reference median (i.e., zero) for WAZ averaged over all ages and for HAZ at age 60 mo. We refer to these measures as the underweight severity and the stunting severity, respectively.

## Results

### GAM Results

The GAM parameters are very similar for boys (Table 1) and girls (Table 1 of File S1), and the statistically significant parameters have the following interpretations. WAZ exhibits global mean reversion (i.e., heavier children tend to lose weight and lighter children tend to gain weight), which is more pronounced in younger children, and local mean reversion (i.e., children who have recently lost weight tend to gain it back and vice versa), which is more pronounced in older children. Taller children tend to gain weight although children who recently grew taller are less likely to gain weight. Current diarrhea status has no impact on weight gain overall, although older boys are more likely to gain weight after a diarrhea episode while younger boys are more apt to lose weight (this effect is not observed in girls). HAZ also exhibits global mean reversion that is more prominent in younger children and local mean reversion that is more prominent in older children, and the magnitude of global mean reversion for HAZ is larger than it is for WAZ. Heavier children are more apt to grow taller, an effect that is more evident in younger children. Current diarrhea status and recent weight gain are not associated with height gain. Diarrhea is reinforcing; i.e., current diarrhea tends to lead to future diarrhea. Lighter children and children who recently gained weight both tend to get more diarrhea, and the former effect is more pronounced in older girls than younger girls. Current height of boys has no overall impact on getting diarrhea, although taller girls are more likely to get diarrhea than shorter girls. Older tall children are more likely to get diarrhea than younger tall children.

**Figure 1:**
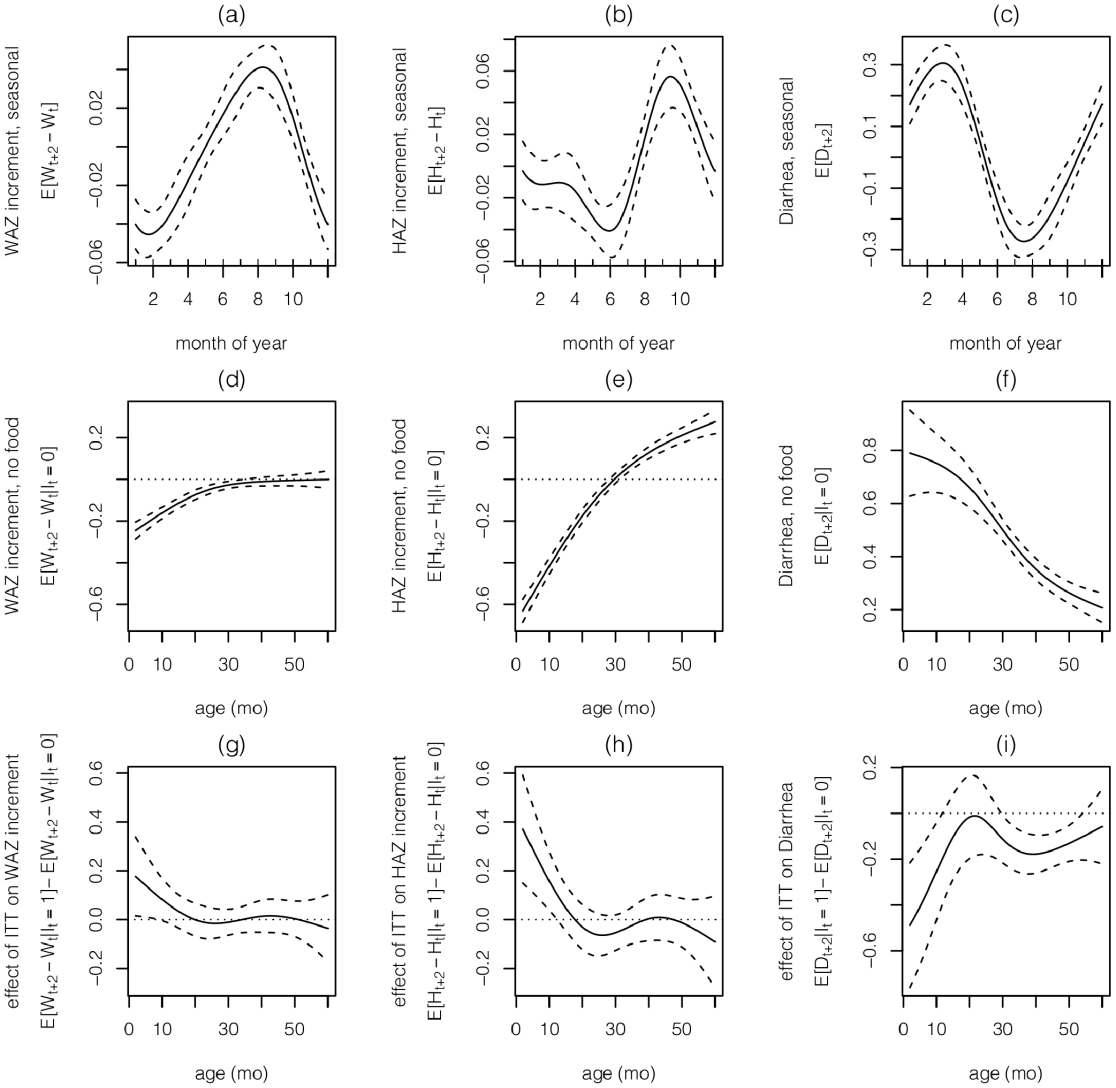
The estimated spline functions (and 95% confidence intervals) for boys from the GAM. The seasonal functions (**a**) 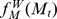, (**b**) 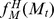 and (**c**) 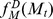 for WAZ and HAZ increments and diarrhea. The age functions in the absence of supplementary food (**d**) 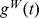, (**e**) 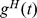 and (**f**) 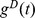 for WAZ and HAZ increments and diarrhea. The age functions due to ITT (**g**) 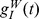, (**h**) 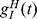 and (**i**) 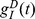
for WAZ and HAZ increments and diarrhea.

Turning to the spline functions, which are also very similar for boys (Fig. 1) and girls (Fig. 1 of File S1), we see that low WAZ and high diarrhea prevalence are on very similar annual cycles (with November - April being the most severe months), while HAZ has a more rapid increase and lags slightly behind WAZ, peaking during August - November (Figs. 1a–c). In the absence of intervention, faltering in WAZ and HAZ occurs during the first 2 yr, at which point WAZ stabilizes and HAZ increases for boys (Figs. 1d–e); for girls, WAZ does not stabilize until 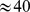 mo and HAZ begins to increase at 30 mo. The prevalence of diarrhea decreases with age (Fig. 1f). The effect of ITT (i.e., supplementary food) has a similar pattern for all 3 variables: the impact is beneficial (i.e., higher WAZ and HAZ, lower diarrhea) and linearly decreasing between 6 and 20 months, and thereafter has little or no effect (with the exception that ITT leads to an increase in diarrhea for older girls). ITT’s effect on HAZ is twice as large as its effect on WAZ.

The covariance parameters are very similar for boys and girls (§1 of File S1), where weight gain and height gain are positively correlated, weight gain and diarrhea are negatively correlated, and height gain and diarrhea are the least correlated. The adjusted *R* ^2^ for 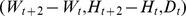 are 0.151, 0.259 and 0.041 for boys, and 0.226, 0.309 and 0.052 for girls.

### Food Allocation Results

**Figure 2:**
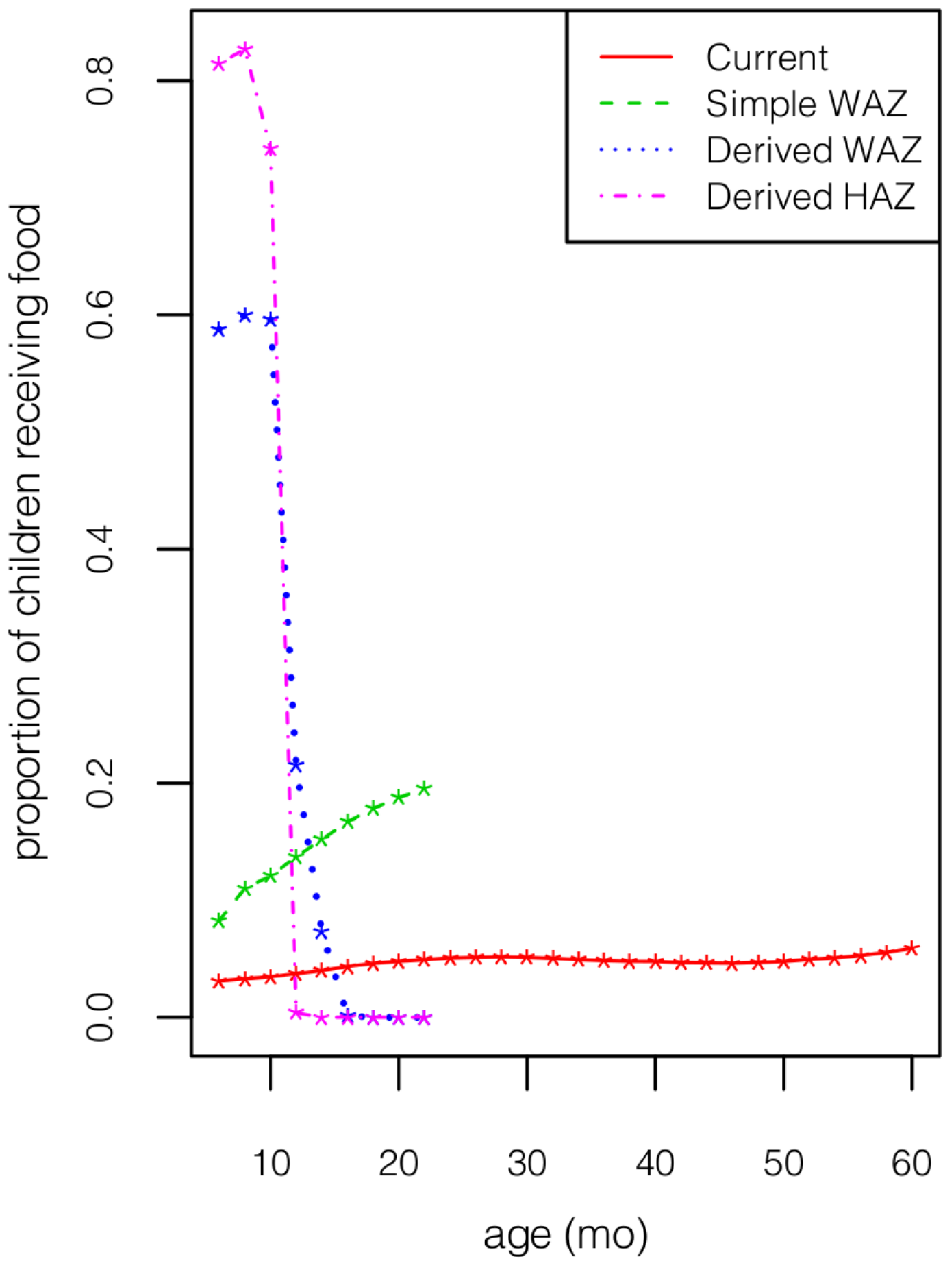
The proportion of boys by age who receive food under the various allocation policies.

**Figure 3:**
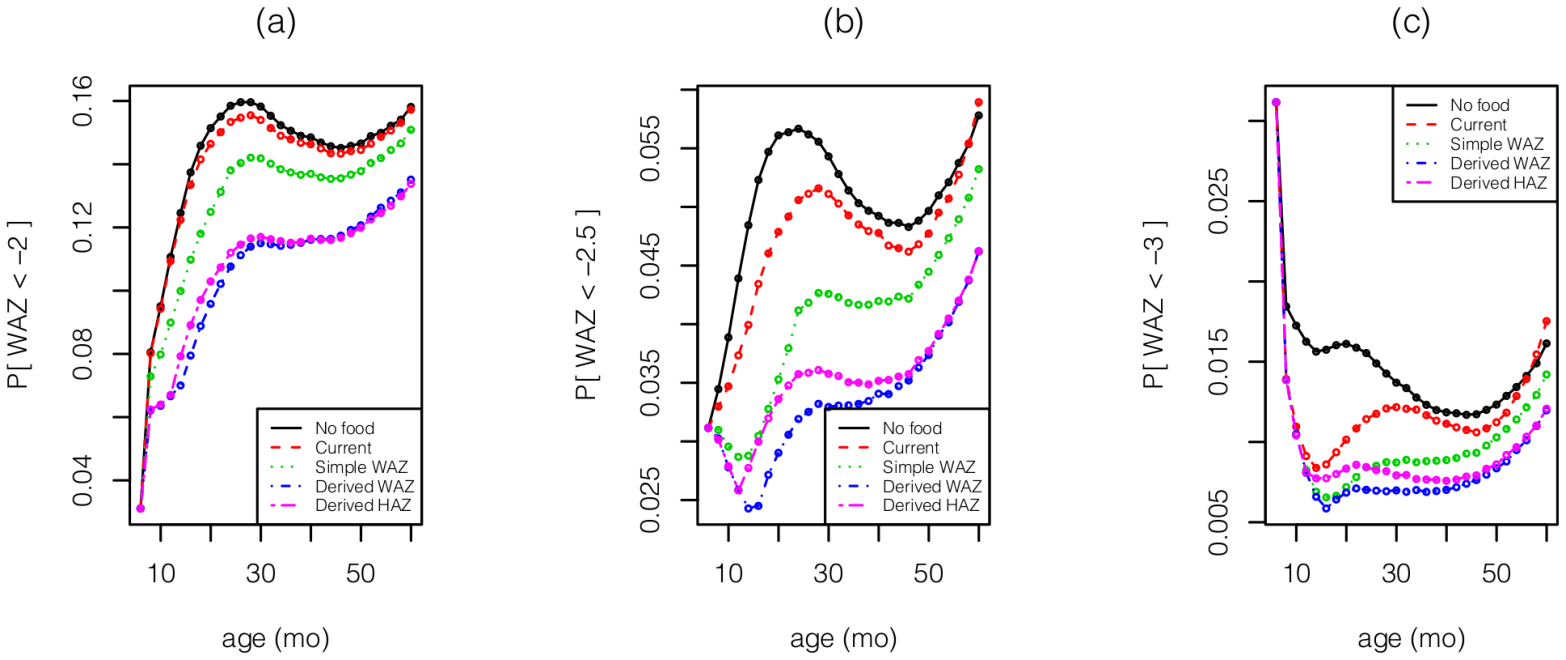
For boys, the left tails, *P*(WAZ <*θ*) for *θ* equals (a) −2, (b) −2.5, (c) −3 vs. age under the various policies.

**Figure 4:**
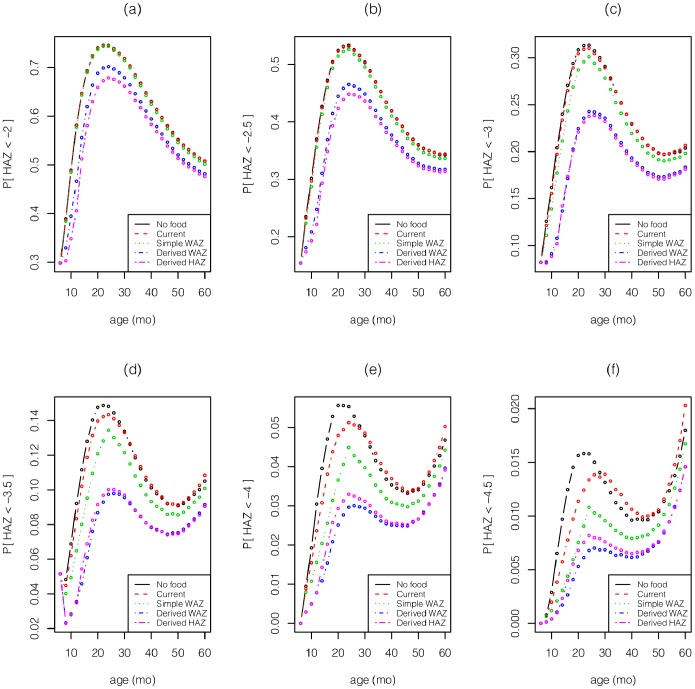
For boys, the left tails, *P*(HAZ <*θ*) for *θ* equals (a) −2, (b) −2.5, (c) −3, (d) −3.5, (e) −4, (f) −4.5 vs. age under the various policies.

**Figure 5:**
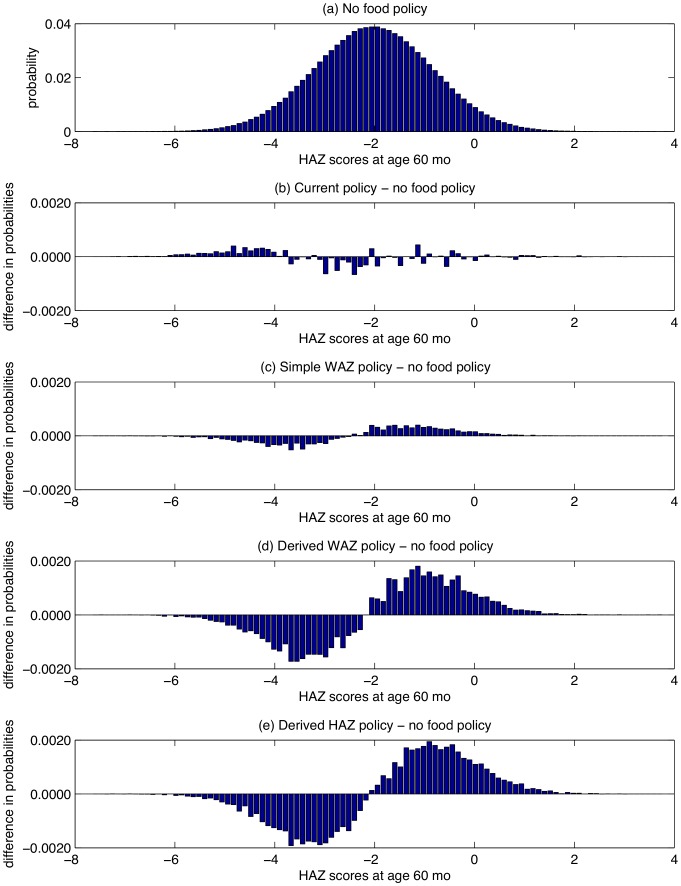
For boys, (a) the PDF for HAZ at age 60 mo, and (b)-(e) the difference in PDFs for HAZ at age 60 mo between 2 policies.

The results for boys (Figs. 2–5) and girls (Figs. 2–5 of File S1) are very similar, aside from the fact that girls are less underweight than boys, and hence have a smaller food budget. Given the strong influence of age on the effect of intervention (Figs. 1g–i), it is not surprising that the two derived policies allocate much of the food to children 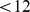 mo (Fig. 2). Consequently, the two derived policies reduce the far left tail of the HAZ and WAZ distributions between 6–12 mo (Figs. 3–4). Although some of this gain is dissipated through the next 4 years, these 2 policies still dominate the other policies throughout the first 5 years. The current policy incurs a significant increase in extreme underweight (WAZ 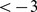) during 12–30 mo because it fails to feed enough children 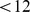 mo who are in danger of faltering. A similar outcome – but at WAZ 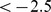
– is incurred by the simple WAZ policy for children during 20–24 mo.

The severity indices (Table 2 for boys, Table 2 of File S1 for girls) reveal that the derived HAZ policy achieves slightly smaller underweight and stunting severity indices than the derived WAZ policy, although both policies perform well on both measures relative to the other policies, and achieve a 13.6–14.1% reduction in WAZ severity and a 7.1–8.0% reduction in HAZ severity relative to the current policy. The performance gap between the 2 derived policies and the simple WAZ policy is 2–3 times larger than the gap between the simple WAZ policy and the no food policy. In our simulations, the current policy increases the HAZ severity index by 1.1% relative to the no food policy for boys, and increases it by 0.1% for girls. An examination of the stunting PDFs for the 5 policies (Fig. 5) reveals that the current policy reduces the number of children with 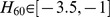, but increases the number of children with 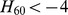. This increase in the extreme tail occurs because there is a small group of children with WAZ values that are consistently 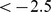 and with very low HAZ levels, and ITT further lowers their HAZ levels during months 18–36 and 46–60 (although even at the lowest points in Fig. 1 h of **−**0.08 at 25 mo and **−**0.1 at 60 mo, children continue to grow taller under ITT). In contrast, the 2 derived policies reduce the number of children with 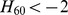 and move them to the 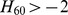 region.

## Sensitivity Analysis

**Figure 6:**
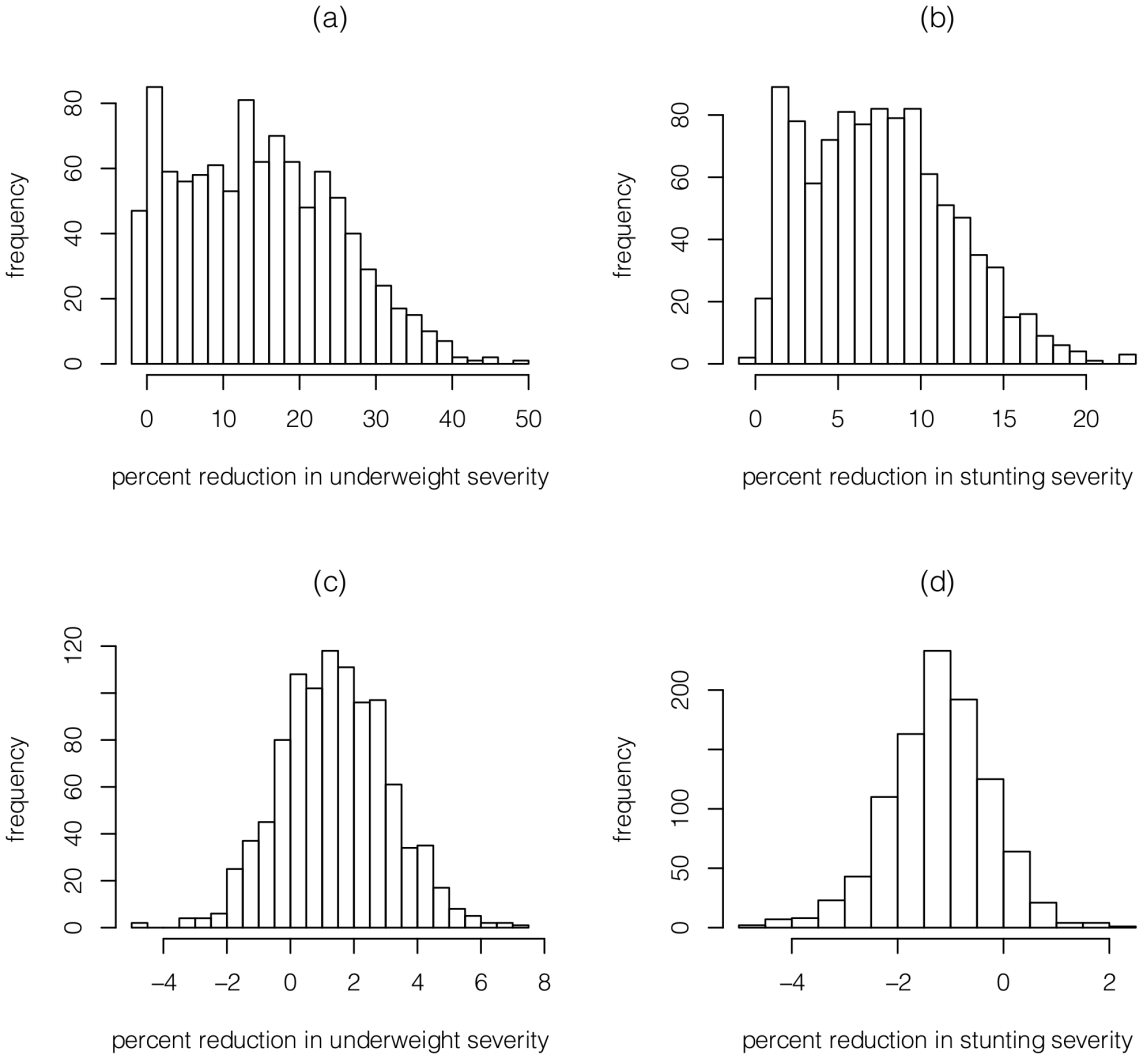
For boys, the PDFs of the percent reduction of the severity indices of (a)-(b) the derived WAZ policy relative to the current policy, and (c)-(d) the current policy relative to the no food policy.

We perform a sensitivity analysis for boys to assess the robustness of our main result that the 2 derived policies outperform the current policy. We perform the following procedure 10^3^ times: take a random sample with replacement of 1047 boys (which is the original sample size in the data set) from our data set, re-estimate the statistical model (i.e., get new values for the parameters in Table 1 and the functions in Fig. 1), use the statistical model to simulate 10^4^ boys under the current policy and the derived WAZ policy (for all runs, the derived WAZ policy is based on the results in Table 1 and Fig. 1, not on the re-estimated model), and compute the percentage reduction in the two severity indices that the derived WAZ policy achieves relative to the current policy. The PDFs of the 10^3^ percentage reductions (Figs. 6a–b) show that the derived WAZ policy achieves a mean percentage reduction of 14.7% for underweight severity and 7.6% for stunting severity relative to the current policy, and the percentage of times that the current policy performs better than the derived WAZ policy is 4.7% for underweight severity and 0.2% for stunting severity.

We perform an identical sensitivity analysis to assess whether the current policy reduces the underweight and stunting severities relative to the no food policy. We find that the current policy reduces underweight severity by 1.4% on average and reduces it 79.7% of the time, whereas the current policy increases stunting severity by 1.2% on average and increases it 90.6% of the time (Figs. 6c–d).

## Discussion

### Statistical Results

The similarity between some of our statistical results and the existing literature is reassuring, and provides some confidence in the remaining statistical results that have no counterpart in the literature. The patterns of growth faltering in Figs. 1d-e are similar to the patterns seen elsewhere [13], as are the decrease in diarrhea prevalence with age (Fig. 1f and [14]). Similarly, the seasonal patterns in Figs. 1a-c are not unlike those found in Malawi [15], and – as is typical in Latin America [16] – stunting is much more prevalent than underweight. Rearranging equations (13)-(16) in the Supporting Material of [4] shows that the model there, which is based on data from Bwamanda in the Democratic Republic of Congo [17], also exhibits global and local mean reversion; global mean reversion in HAZ data is also observed in [15].

Turning to the cross terms, we find that high weight leads to a future increase in HAZ, but that recent weight gain does not; the latter finding agrees with correlations derived in [15]. An analysis of WHZ (rather than WAZ) and HAZ find that both low WHZ and a recent drop in WHZ lead to future reductions in HAZ [18].

Our statistical results provide limited evidence to support the fact that diarrhea can reduce nutritional status. The lack of significance of the *D_t_* coefficient in predicting future WAZ or HAZ gain may be because most of the catch-up growth occurs before the next visit. In addition, the coefficient of 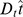 in the WAZ equation for boys is consistent with the observation that infants take longer to catch up in weight than older children [19]. Although our model predicts that underweight children are more apt to get diarrhea, which is consistent with earlier results [20], [21], the same is not true of stunted children (in fact, taller girls are more likely to get diarrhea than shorter girls in our model); some of the anticipated effect (i.e., stunted children are more likely to get diarrhea) may be partially masked by the age dependence (i.e., the value of the 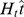 coefficient) and the 2-mo follow-up period. In any case, diarrhea is very difficult to predict and the only factors that play a strong role are seasonality, being underweight, and currently having diarrhea, which collectively suggest environmental and microbiome causes.

Our most intriguing statistical result is the strong dependence of age on the impact of intervention (Figs. 1g–i). Considerable evidence has been amassed about the importance of nutrition in the first 1000 days (from conception to 24 mo) [1], [22], and Figs. 1g-i can be viewed as a refinement of this principle. Our age-dependent results are similar to those in an earlier study in Guatemala [23], which found that – after controlling for diarrhea, previous height and weight, sex, home diet and socioeconomic status – height gain and weight gain due to supplemental food decreased throughout the first 3 years of life and had little effect after age 3. A meta-analysis in Web Appendix 4 of [24] suggests that linear growth during 6–12 mo is larger than during 12–18 mo in populations with average per capita income 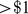/day, but is smaller for incomes 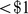/day. Fig. 1 h is not inconsistent with this observation because the income in Guatemala is 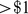/day.

The magnitude of the seasonality in Figs. 1a-b and of the global and local mean reversion (Table 1) implies that approaches that ignore these factors can lead to misleading estimates – and systematic overestimates in the case of global mean reversion – of the increase in weight and height that is due to supplementary feeding.

### Food Allocation Results

The 2 derived policies anticipate growth faltering and exploit the age dependence in the impact of intervention by front-loading much of the intervention on infants (Fig. 2), who gain a substantial head start relative to the current policy (Figs. 3–4). Although some of the gap disappears by the time children reach age 5 years (perhaps partly due to global mean reversion), much of the improvement remains intact at age 5. The derived policies are fairly simple to implement, although they do require knowledge of a child’s height and weight from his previous visit.

Due to the very limited amount of food distributed by the current policy (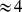
% of children receive food at any point in time) and the fact that many more children are stunted than underweight, the improvements achieved by the 2 derived policies are modest when measured by stunting (HAZ 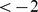), but are more pronounced for severe stunting (HAZ 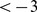) and in the extreme left tail of the HAZ distribution (Fig. 4).

Because of the reinforcing cross terms – i.e., large HAZ leads to future increases in WAZ and large WAZ leads to future increases in HAZ – and the similar age dependence in how intervention increases WAZ and HAZ, the 2 derived policies have very similar performance with respect to reducing the severity of underweight and stunting.

There has been increasing focus on the lifetime effects of stunting [25], and in Latin America, where severe wasting is rare and stunting is prevalent, reducing stunting is one of the primary goals of supplementary feeding programs. To this end, we note that our model predicts that the current policy – because it allows some underweight, stunted children to become very severely stunted (Fig. 5b) – has a slightly higher severity of stunting than the no food policy (Fig. 6d). This result is driven by the fact that ITT leads to a slight decrease in HAZ in our model during months 18–36 and 46–60 (Fig. 1h); it is not clear whether this effect is real (albeit small), or is caused by overshooting of the cubic spline in Fig. 1h. In contrast, despite the wide confidence intervals in Figs. 1g–i, our sensitivity analysis (Figs. 6a–b) suggests that our main result – i.e., that the 2 derived policies outperform the current policy – is somewhat robust.

There has also been concern about the long-term cardiovascular risks associated with catch-up growth in early childhood [26]. While this remains an important issue, particularly for rapid weight gain among infants born at low birth weight, the immediate mortality and developmental risks associated with serious childhood malnutrition would seem to warrant monitored nutritional supplementation.

### Limitations of Analysis

Aside from the obvious shortcoming that our analysis is based on data from a non-randomized study, there are 2 major limitations in our data set. The first is that health promoters in the Guatemala nutrition program had discretion to feed sick children, and hence ITT did not exactly coincide with the actual receipt of food. We use ITT so as not to be biased by the behavior of health promoters. Note that if the health promoters were not given discretion to feed sick children, there may have been an incentive to alter WAZ data (e.g., change **−**2.3 to **−**2.6 so as to feed a sick child). The second limitation in our data set is the amount of missing data. While children were supposed to see a health promoter every 2 mo, the actual time between consecutive visits in the data set was 1 mo, 2 mo, 3 mo, 4 mo and 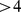
mo with probabilities 0.094, 0.638, 0.122, 0.121 and 0.024, respectively. With respect to estimating the impact of ITT and its age interaction, the primary concern with our assumption that consecutive visits are exactly 2 mo is if ITT causes children to visit more or less frequently relative to no ITT. To assess this issue, we re-ran the GAM with the same predictor variables as before but now with the time until the next visit as the response variable. The overall mean effect of ITT was very close to zero (see Fig. 6 of File S1, which is the analogue to Figs. 1g-i in the main text) and the 3 p-values for the cubic spline were 0.91, 0.88 an 0.96.

There are other aspects that could be investigated: estimating the variance of ITT, the interaction of diarrhea and z scores (e.g., a 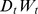 interaction term could address whether diarrhea lowers future weight gain only when current WAZ is low [19]), and whether ITT is a function of *D_ t_* [19]. However, we note that our qualitative conclusions would not change even if diarrhea was omitted from the model. Also, the data used here are not well suited to estimate the influence that pre-intervention (WAZ,HAZ) levels have on the impact of intervention because of the lack of intervention-free children with low WAZ and because of the presence of global mean reversion, which may have a similar effect (Fig. 2 in [27]).

The age dependence in the impact of treatment should not be extrapolated to other settings, other doses or other undernutrition thresholds without performing similar statistical analyses of other longitudinal data sets. It should be noted that the community health system within which the nutritional surveillance and supplementation program is embedded also provides basic preventive and therapeutic health services, including routine antihelminthic medication for children greater than 2 years of age and when needed, referral to local and regional, clinical facilities. In addition, identification of malnutrition and the provision of supplementation may elevate the nutritional needs of the child in question and result indirectly in a relative reallocation of some family resources to the child. This in turn could modify the impact of supplementation on growth in different cultural or resource settings. It may be the case that 100 kCal/day is an insufficient dose – particularly after taking into account the possibility of food sharing within families – to improve the nutritional status of children over 2 years old, but that larger doses are sufficient. Similarly, even at the current dose, our findings should not be extrapolated to thresholds other than WAZ = −2.5. It is possible that age-specific treatment effects differ by level of undernutrition. In particular, children 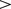
20 mo may respond more to supplementary feeding when their initial undernutrition levels are more severe, because of the developmental delays associated with undernutrition. If so, regression discontinuity around a more extreme threshold might have yielded flatter estimated age functions in Figs. 1g–i.

### Conclusion

Fitting a trivariate statistical model of WAZ, HAZ and diarrhea to a longitudinal data set from a nutritional program in Guatemala generates results that reinforce and quantify relationships that are already known, and identify new relationships, including a strong age dependence in the impact of supplementary food on WAZ, HAZ and diarrhea, with the benefits decreasing from 6 mo to 20 mo old and staying at a small level between 20 and 60 mo old. Our statistical results highlight the danger of ignoring seasonality and global and local mean reversion when estimating the impact of supplementary food. The food allocation policies derived here perform better than the current policy: by allocating much of the food to infants, the derived policies reduce the underweight severity by 13.6–14.1% and the stunting severity by 7.1–8.0% relative to the current policy, despite using a budget that allocates food to only 4.7% of the children at any time under the current policy. This result should not be extrapolated to other geographical regions, to doses other than 100 kCal/day, or to undernutrition thresholds other than WAZ = −2.5. To confirm this result, the derived policies should be compared to the current policy or the simple WAZ policy in a randomized study. More generally, given the paucity of randomized controlled trials with intervention-free control groups, our statistical approach has the potential to uncover new knowledge from observational studies pertaining to therapeutic and supplementary food for children, and our optimization approach allows this knowledge to be leveraged to improve nutritional status in a budget-constrained setting.

## Supporting Information

File S1 Supporting Material. Gives the regression model equations, the derivation of the proposed food allocation policies, and the results for girls.
(PDF)
