# Supplementary material for: Quantifying and Exploiting the Age Dependence in the Effect of Supplementary Food for Child Undernutrition
Source: PLoS One. 2014 Jun 26;9(6):e99632. doi: 10.1371/journal.pone.0099632 (PMC4072673; doi:10.1371/journal.pone.0099632)
Supplement: File S1 — Supporting Material. Gives the regression model equations, the derivation of the proposed food allocation policies, and the results for girls. (PDF) [file pone.0099632.s001.pdf]

SUPPORTING MATERIAL

The GAM is given in §1 and the derived allocation policies are constructed in §2. Tables 1-2 and Figs. 1-5, which are the results for girls, and Fig. 6, which contains the results of a sensitivity analysis, are referred to in the main text.

1 Regression Model

With the parameter estimates included, the GAM equations for boys are given by

$$\begin{aligned} E[W_{t+2} - W_t] =& -0.230 - 0.095W_t + 0.014W_t\hat{t} - 0.256(W_t - W_{t-2}) - 0.090(W_t - W_{t-2})\hat{t} \\ & + 0.038H_t + 0.006H_t\hat{t} - 0.020(H_t - H_{t-2}) - 0.007(H_t - H_{t-2})\hat{t} \\ & + 0.003D_t + 0.014D_t\hat{t} + f_M^W(M_t) + g^W(t) + g_I^W(t)I_t, \end{aligned} \tag{1}$$

$$\begin{aligned} E[H_{t+2} - H_t] =& -0.907 + 0.124W_t - 0.068W_t\hat{t} - 0.000(W_t - W_{t-2}) + 0.021(W_t - W_{t-2})\hat{t} \\ & - 0.177H_t + 0.092H_t\hat{t} - 0.205(H_t - H_{t-2}) - 0.022(H_t - H_{t-2})\hat{t} \\ & - 0.003D_t + 0.007D_t\hat{t} + f_M^H(M_t) + g^H(t) + g_I^H(t)I_t, \end{aligned} \tag{2}$$

$$\begin{aligned} \ln E[D_{t+2}] =& -0.751 - 0.184W_t - 0.056W_t\hat{t} + 0.183(W_t - W_{t-2}) + 0.084(W_t - W_{t-2})\hat{t} \\ & - 0.007H_t + 0.068H_t\hat{t} + 0.063(H_t - H_{t-2}) - 0.041(H_t - H_{t-2})\hat{t} \\ & + 0.102D_t - 0.021D_t\hat{t} + f_M^D(M_t) + g^D(t) + g_I^D(t)I_t. \end{aligned} \tag{3}$$

The sample covariance matrix calculated from the residuals of equations (1)-(3) is

$$\Sigma = \begin{pmatrix} 0.10 & 0.03 & -0.04 \\ 0.03 & 0.19 & 0.01 \\ -0.04 & 0.01 & 1.54 \end{pmatrix}. \tag{4}$$

The GAM equations for girls are

$$
\begin{aligned}
E[W_{t+2} - W_t] = &- 0.178 - 0.101 W_t + 0.029 W_t \hat{t} - 0.249(W_t - W_{t-2}) - 0.071(W_t - W_{t-2})\hat{t} \\
&+ 0.033 H_t + 0.004 H_t \hat{t} - 0.006(H_t - H_{t-2}) + 0.019(H_t - H_{t-2})\hat{t} \\
&+ 0.003 D_t + 0.002 D_t \hat{t} + f_M^W(M_t) + g^W(t) + g_I^W(t) I_t, \qquad (5)
\end{aligned}
$$

$$
\begin{aligned}
E[H_{t+2} - H_t] = &- 0.765 + 0.118 W_t - 0.070 W_t \hat{t} + 0.003(W_t - W_{t-2}) - 0.009(W_t - W_{t-2})\hat{t} \\
&- 0.188 H_t + 0.106 H_t \hat{t} - 0.193(H_t - H_{t-2}) - 0.029(H_t - H_{t-2})\hat{t} \\
&- 0.003 D_t + 0.006 D_t \hat{t} + f_M^H(M_t) + g^H(t) + g_I^H(t) I_t, \qquad (6)
\end{aligned}
$$

$$
\begin{aligned}
\ln E[D_{t+2}] = &- 0.660 - 0.232 W_t - 0.092 W_t \hat{t} + 0.148(W_t - W_{t-2}) + 0.073(W_t - W_{t-2})\hat{t} \\
&+ 0.115 H_t + 0.068 H_t \hat{t} + 0.032(H_t - H_{t-2}) + 0.060(H_t - H_{t-2})\hat{t} \\
&+ 0.132 D_t + 0.017 D_t \hat{t} + f_M^D(M_t) + g^D(t) + g_I^D(t) I_t. \qquad (7)
\end{aligned}
$$

The sample covariance matrix calculated from the residuals of equations (5)-(7) is

$$
\Sigma = \begin{pmatrix} 0.10 & 0.03 & -0.05 \\ 0.03 & 0.19 & 0.00 \\ -0.05 & 0.00 & 1.48 \end{pmatrix}. \qquad (8)
$$

2 The Derived Allocation Policies

To construct a policy that is easy to use and understand, we make 4 simplifying assumptions. First, we restrict ourselves to myopic policies, i.e., policies that consider only WAZ severity 2 mo in advance (all our arguments in this section use WAZ although they also hold if we replace WAZ by HAZ). Suppose we are making food allocation decisions at time τ. We have N children indexed by $i = 1, \ldots, N$, and let $W_{i,\tau+2}$ be child i's WAZ score at time $\tau + 2$ and

let the decision variables be $I_{i\tau} = 1$ if child i receives food at time τ and $I_{i\tau} = 0$ otherwise. At time τ and conditional on our decision $I_{i\tau}$, this child's expected contribution to the WAZ severity at time $\tau + 2$ is $E[W^2_{i,\tau+2}|I_{i\tau}]I_{\{W_{i,\tau+2}<0\}}$. We make 2 more assumptions to simplify this expression: we replace $E[W^2_{i,\tau+2}|I_{i\tau}]$ by $E[W_{i,\tau+2}|I_{i\tau}]^2$ (or, equivalently, we assume that there is no randomness in predicting $W_{i,\tau+2}|I_{i\tau}$ at time τ) and we assume that children's WAZ scores do not change sign between times τ and $\tau + 2$. While this latter assumption may be violated, it only impacts our policy if the food budget B is sufficiently large that children with WAZ near 0 might receive food (which is not the case in our setting or in most practical settings). These 2 assumptions allow us to approximate $E[W^2_{i,\tau+2}|I_{i\tau}]I_{\{W_{i,\tau+2}<0\}}$ by $E[W_{i,\tau+2}|I_{i\tau}]^2 I_{\{W_{i\tau}<0\}}$. Our fourth assumption will be introduced later.

Let $c_{i\tau} = 0.5$ if child i's age is $\in [6, 12)$ mo at time τ and $c_{i\tau} = 1$ if child i's age is $\in [12, 60]$ mo at time τ. Then our optimization problem at time τ is

$$\min_{I_{i\tau}\in\{0,1\}} \quad \sum_{i=1}^{N} E[W_{i,\tau+2}|I_{i\tau}]^2 I_{\{W_{i\tau}\}<0} \tag{9}$$

$$\text{subject to} \quad \sum_{i=1}^{N} c_{i\tau} I_{i\tau} \leq B. \tag{10}$$

Define $\delta_{i\tau} = (E[W_{i,\tau+2}|I_{i\tau} = 1]^2 - E[W_{i,\tau+2}|I_{i\tau} = 0]^2)I_{\{W_{i\tau}\}<0}$, which is the change in child i's contribution to WAZ severity due to food, where the children with more negative values generate a greater reduction in severity. If c_{it} equaled 1 for all i and τ, then the optimal solution to (9)-(10) would rank children by δ_{it} and give food to the B children with the lowest $\delta_{i\tau}$ values; this can be shown with a simple interchange argument (i.e., a proof by contradiction, where one assumes that a different child with a higher value of $\delta_{i\tau}$ receives some food instead of a child with a lower value of $\delta_{i\tau}$). However, because $c_{i\tau} = 0.5$ for children under 12 mo, it is optimal to make 2 lists of children ranked by $\delta_{i\tau}$, where list A has children with $c_{i\tau} = 0.5$ and list B has children with $c_{i\tau} = 1$, and then allocate the food by comparing the sum of the $\delta_{i\tau}$ values of the next 2 children in list A to the $\delta_{i\tau}$ value of the next child in list B; see Algorithm 1 for details.

Algorithm 1 Optimal policy

Set $I_{i\tau} = 0 \, \forall \, i$ and total amount of food given = 0
Create two lists of children, A = $\{i : c_{i\tau} = 0.5\}$ and B = $\{i : c_{i\tau} = 1\}$
Sort A and B in the increasing order of $\delta_{i\tau}$, and let A(1), A(2) and B(1) denote the first two children in list A and the first child in list B
while total amount of food allocated is less than B **do**
  if $\delta_{\mathrm{A}(1)\tau} + \delta_{\mathrm{A}(2)\tau} > \delta_{\mathrm{B}(1)\tau}$ **then**
    set $I_{\mathrm{B}(1)\tau} = 1$; i.e., give food to the first child in list B
    increase the total amount of food allocated by 1
  else
    set $I_{\mathrm{A}(1)\tau} = 1$; i.e., give food to the first child in list A
    increase the total amount of food allocated by 0.5
  end if
  Delete the child who received food from the list
end while
if total amount of food allocated is greater than B **then**
  Instead of the last child who got food (who is from list B), give food to the first child in list A
end if

Algorithm 1 is awkward to implement. Hence, we introduce our fourth assumption, which is that N is large enough so that the WAZ scores are dense in the real line and its distribution is independent of age. It follows that the heuristic policy (see Algorithm 2), which simply ranks all children by $\delta_{i\tau}/c_{i\tau}$ and allocates the food to children with smaller (i.e., more negative) values, achieves nearly the same performance as the optimal solution.

Algorithm 2 Heuristic policy

Set $I_{i\tau} = 0$ for all i, total amount of food given = 0
Sort all the children in the increasing order of $\delta_{i\tau}/c_{i\tau}$
Let $n = \max\{j \mid \sum_{i=1}^{j} c_{i\tau} \leq B\}$
Set $I_{i\tau} = 1 \;\; \forall \, i \leq n$

Tables

Table 1: The estimated parameters for girls from the GAM

		Estimating $W_{t+2} - W_t$			Estimating $H_{t+2} - H_t$			Estimating D_{t+2}		
Variable	Description	Est.	S.E.	p	Est.	S.E.	p	Est.	S.E.	p
	Intercept	-0.178	0.018	0.000	-0.765	0.024	0.000	-0.660	0.075	0.000
W_t	WAZ	-0.101	0.006	0.000	0.118	0.009	0.000	-0.231	0.039	0.000
$W_t\hat{t}$	WAZ×age	0.029	0.006	0.000	-0.070	0.008	0.000	-0.092	0.035	0.009
$W_t - W_{t-2}$	WAZ increment	-0.249	0.011	0.000	0.003	0.015	0.840	0.148	0.067	0.026
$(W_t - W_{t-2})\hat{t}$	WAZ incr.×age	-0.071	0.010	0.000	-0.009	0.013	0.518	0.073	0.055	0.188
H_t	HAZ	0.033	0.005	0.000	-0.188	0.007	0.000	0.115	0.032	0.000
$H_t\hat{t}$	HAZ×age	0.004	0.005	0.376	0.106	0.007	0.000	0.068	0.029	0.019
$H_t - H_{t-2}$	HAZ increment	-0.006	0.009	0.514	-0.193	0.012	0.000	0.032	0.052	0.540
$(H_t - H_{t-2})\hat{t}$	HAZ incr.×age	0.019	0.007	0.008	-0.029	0.010	0.003	0.060	0.040	0.136
D_t	Diarrhea	0.003	0.003	0.335	-0.003	0.004	0.399	0.132	0.012	0.000
$D_t\hat{t}$	Diarrhea×age	0.002	0.003	0.549	0.006	0.004	0.121	0.017	0.012	0.157

For each variable, we give the estimated coefficient (Est.), the standard error (S.E.) and the p-value.

Table 2: Food allocation policies

Name of Policy	Children Receiving Food	Severity Index	
		Underweight	Stunting
No Food	None	1.62	5.83
Current	$W_t < -2.5$	1.60	5.84
Simple WAZ	Under 2 yr and prioritized by W_t	1.55	5.76
Derived WAZ	Prioritized by $(E[W_{t+2}\|I_t = 1)]^2 - E[W_{t+2}\|I_t = 0]^2)/c_t$	1.45	5.64
Derived HAZ	Prioritized by $(E[H_{t+2}\|I_t = 1)]^2 - E[H_{t+2}\|I_t = 0]^2)/c_t$	1.44	5.62

A description of the food allocation policies and their severity indices for girls, which measure the average of squared shortfalls below the reference median (i.e., zero) for WAZ averaged over all 28 measured ages (underweight) and for HAZ at age 60 mo (stunting).

Figures

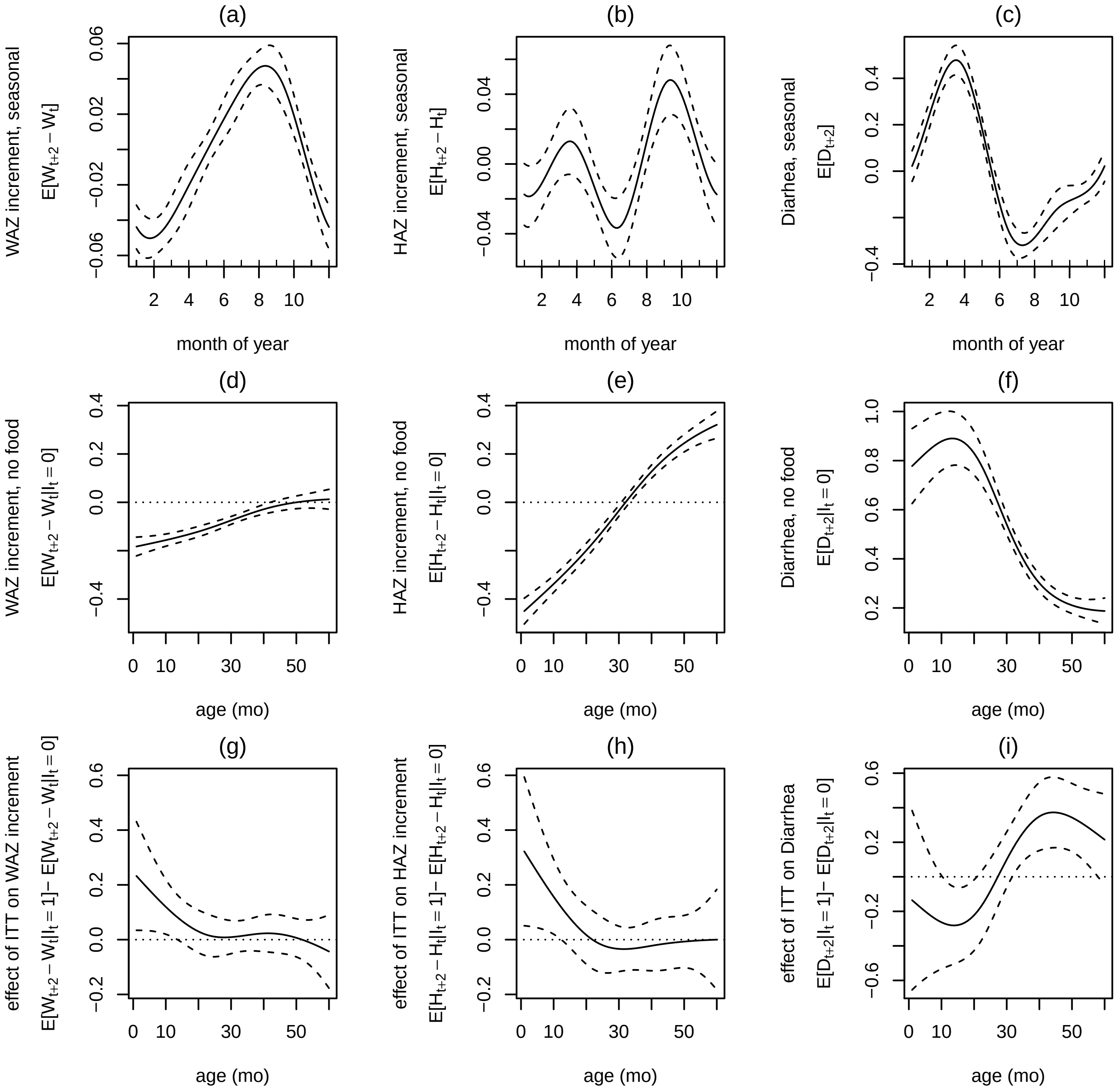

Figure 1: The estimated spline functions (and 95% confidence intervals) for girls from the GAM. The seasonal functions **(a)** $f_M^W(M_t)$, **(b)** $f_M^H(M_t)$ and **(c)** $f_M^D(M_t)$ for WAZ and HAZ increments and diarrhea. The age functions in the absence of supplementary food **(d)** $g^W(t)$, **(e)** $g^H(t)$ and **(f)** $g^D(t)$ for WAZ and HAZ increments and diarrhea. The age functions due to ITT **(g)** $g_I^W(t)$, **(h)** $g_I^H(t)$ and **(i)** $g_I^D(t)$ for WAZ and HAZ increments and diarrhea.

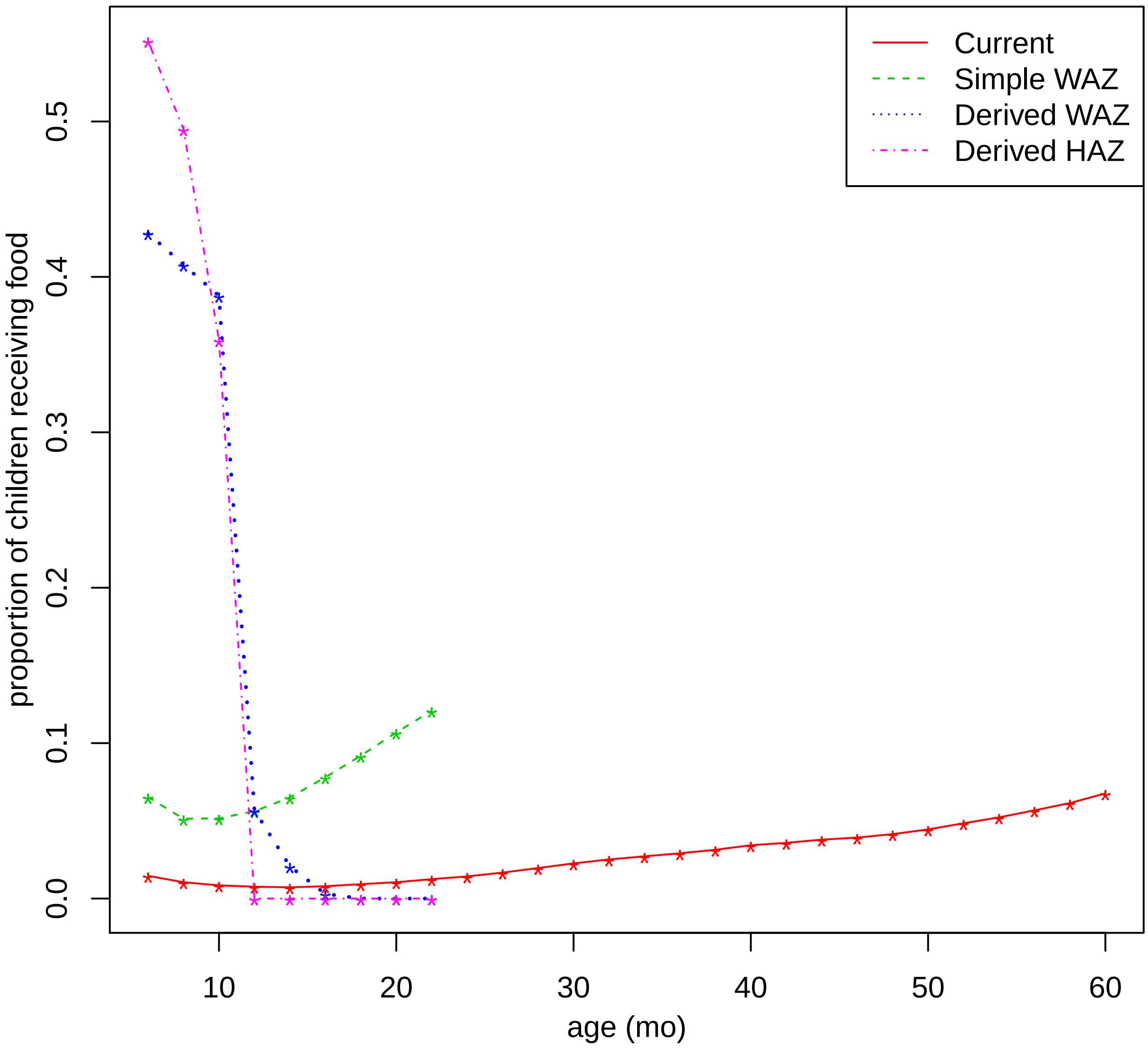

Figure 2: The proportion of girls by age who receive food under the various allocation policies.

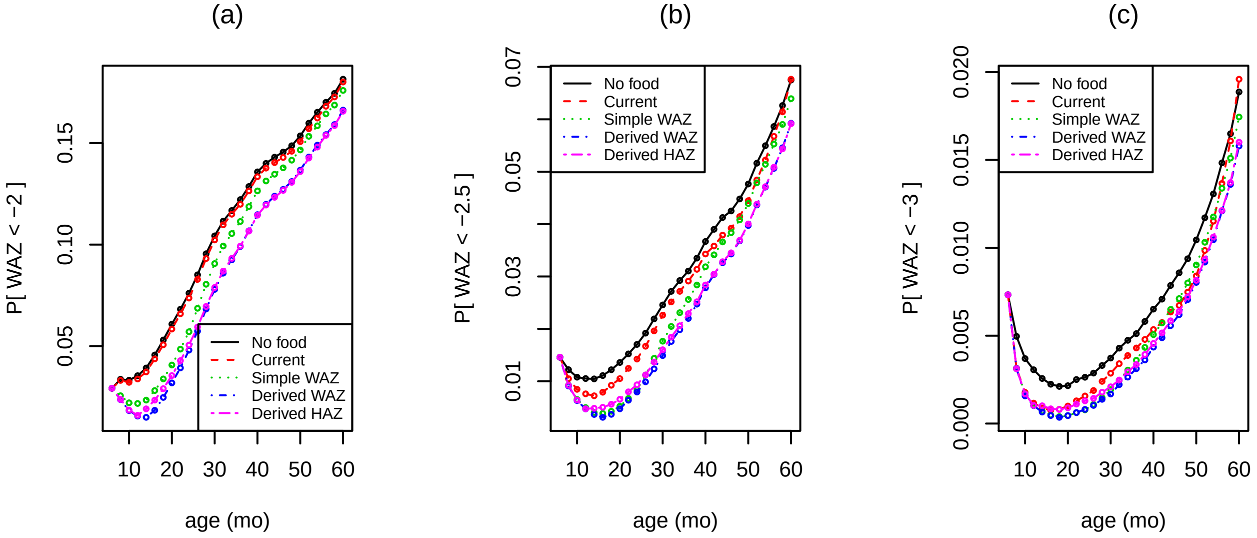

Figure 3: For girls, the left tails, $P(\text{WAZ}< \theta)$ for θ equals **(a)** -2, **(b)** -2.5, **(c)** -3 vs. age under the various policies.

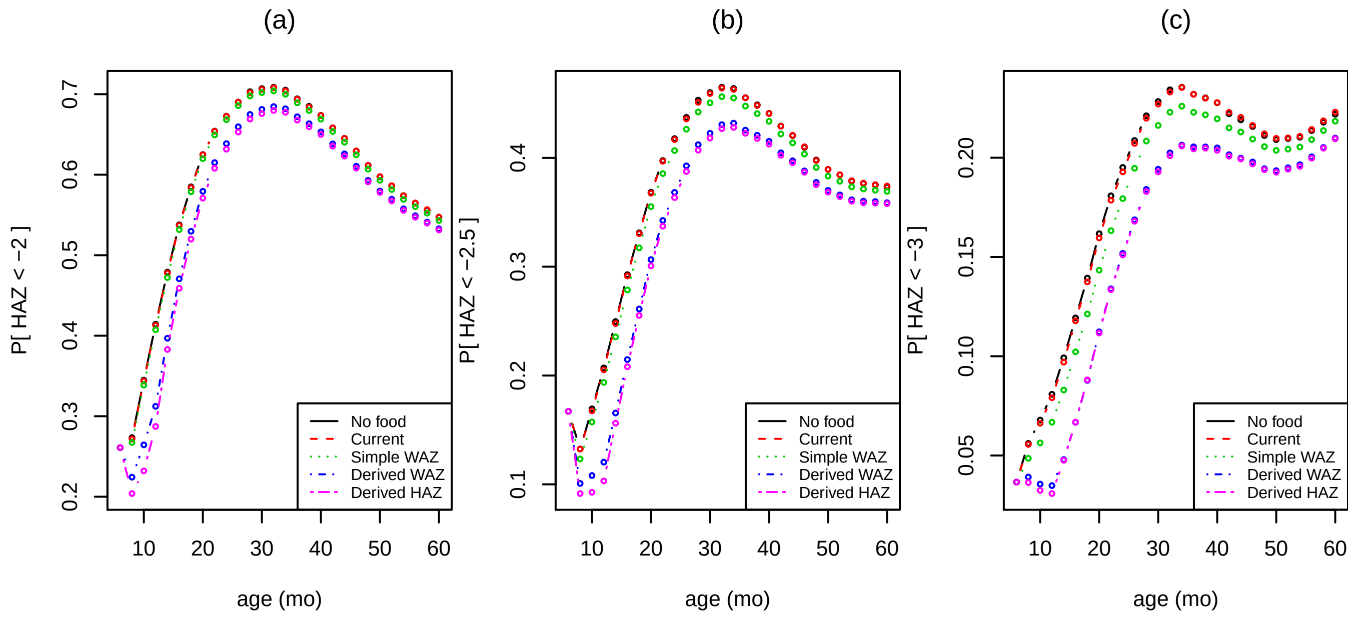

Figure 4: For girls, the left tails, $P(\text{HAZ}< \theta)$ for θ equals **(a)** -2, **(b)** -2.5, **(c)** -3, **(d)** -3.5, **(e)** -4, **(f)** -4.5 vs. age under the various policies.

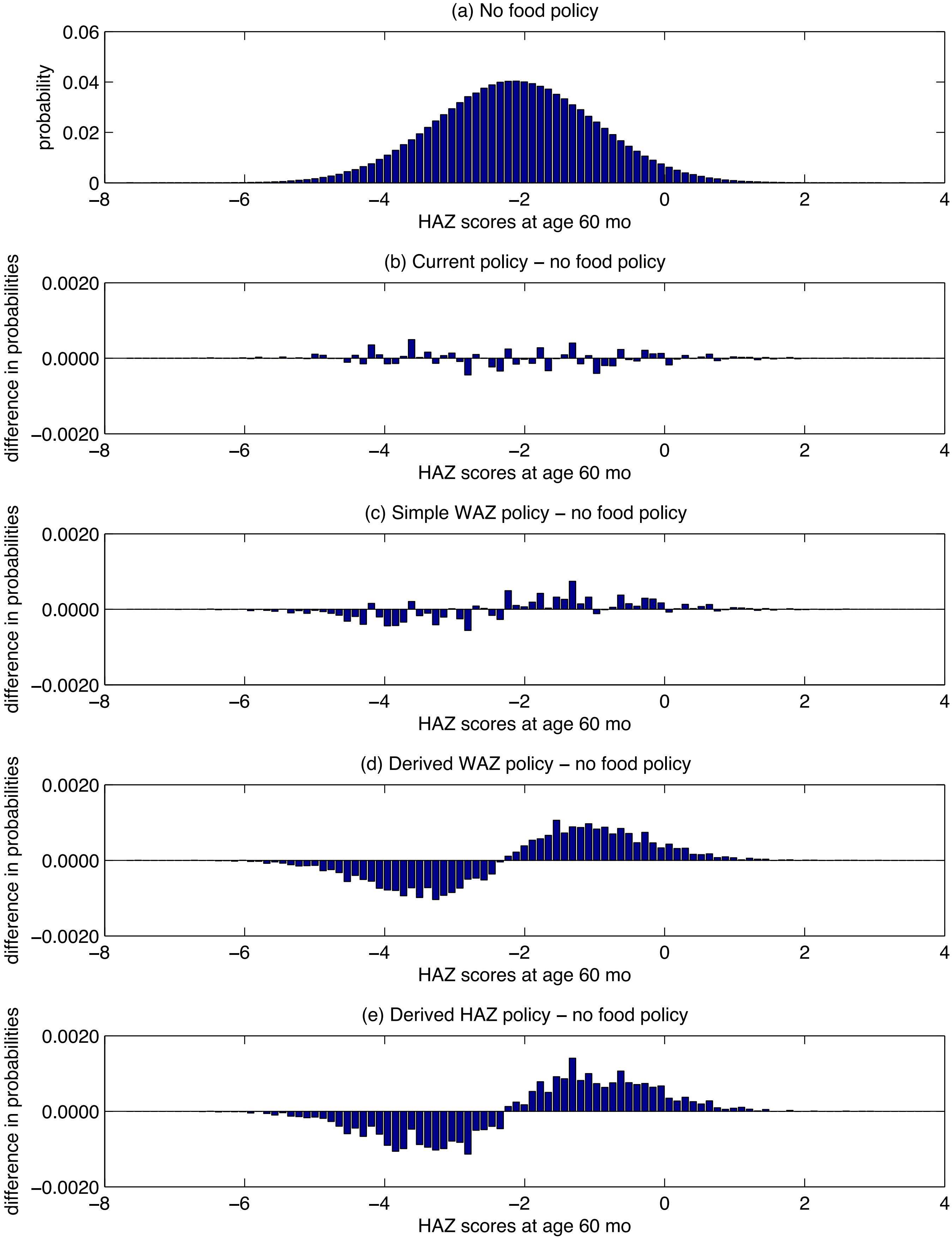

Figure 5: For girls, **(a)** the PDF for HAZ at age 60 mo, and **(b)-(e)** the difference in PDFs for HAZ at age 60 mo between 2 policies.

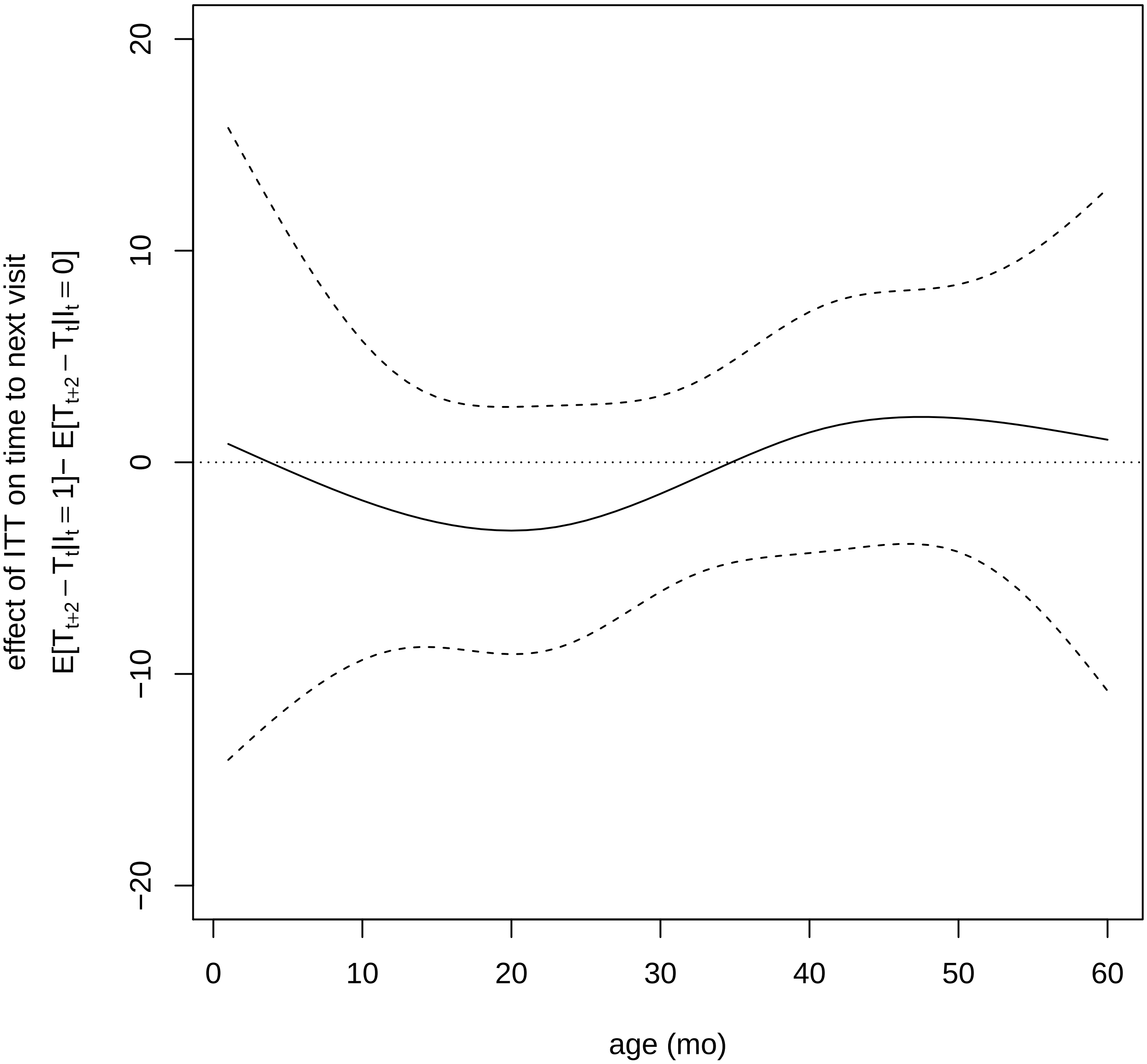

Figure 6: For boys, the estimated spline function (and 95% confidence interval) for the age-dependent impact of ITT in the GAM, where the response variable is the time until the next visit for a child of age t mo (T_t).
